# Subjective correlates of stress management in outpatient cardiac rehabilitation: the predictive role of perceived heart risk factors

**DOI:** 10.15171/jcvtr.2018.16

**Published:** 2018-06-29

**Authors:** Saeid Komasi, Ali Soroush, Mozhgan Saeidi, Agostino Brugnera, Massimo Rabboni, Mario Fulcheri, Danilo Carrozzino, Paolo Marchettini, Angelo Compare

**Affiliations:** ^1^Clinical Research Development Center, Imam Reza Hospital, Kermanshah University of Medical Sciences, Kermanshah, Iran; ^2^Lifestyle Modification Research Center, Imam Reza Hospital, Kermanshah University of Medical Sciences, Kermanshah, Iran; ^3^Cardiac Rehabilitation Center, Imam Ali Hospital, Kermanshah University of Medical Sciences, Kermanshah, Iran; ^4^Department of Human and Social Sciences, University of Bergamo, Bergamo, Italy; ^5^2th Psychiatry Unit, Papa Giovanni XXIII Hospital, Bergamo, Italy; ^6^Department of Psychological, Health, and Territorial Sciences, University “G. d’Annunzio” of Chieti-Pescara, Chieti, Italy; ^7^Department of Neurology, San Raffaele Hospital Milano and Pain Center, Centro Diagnostico Italiano, Milan, Italy

**Keywords:** Cardiovascular Disease, Psychological Stress, Risk Factors, Rehabilitation

## Abstract

***Introduction:*** The causal attributions and perceived risk factors can affect patients’ health behaviors. Therefore, the present study aimed to assess (i) the effect of an outpatient cardiac rehabilitation (CR) program on perceived heart risk factors (PHRFs) and on psychological stress, and (ii) the role of changes of PHRFs at pre-post CR in predicting changes in psychological stress.

***Methods:*** In this longitudinal study, 110 CR patients were assessed from June to November 2016 in a hospital in Iran. Perceived heart risk factors and perceived stress were investigated using the PHRFs scale and the Depression, Anxiety, Stress Scale-21, respectively. PHRFs and DASS-21 Stress scale scores were compared before and after 26 sessions of exercise-based CR through paired sample t-tests. In addition, we investigated the effect of PHRF’s change scores on DASS-21 Stress scale scores using linear regression analysis.

***Results:*** Results showed that CR has a little impact in improving the patients’ perception of heart risk factors, However, CR is significantly effective in reducing stress (*P* < 0.05). Regression analysis evidenced that improvements in patients’ perception of risk factors can significantly predict a reduction in psychological stress (P = 0.030). The model explained 11.2% of the variance in the results.

***Conclusion:*** PHRFs appear to be significant predictive components of CR’s stress reduction. Practitioners should focus on patients’ perception of risk factors to facilitate stress management in CR program.

## Introduction

Stress and anxiety are considered one of the most important risk factors for the development of cardiac diseases.^1-4^ Past reports evidenced that stress reduction programs are associated with positive cardiac outcomes,^5-7^ so that the management and control of perceived stress are actually emphasized during the acute phase and the subsequent cardiac rehabilitation (CR) of cardiovascular patients.^2,5,6^ CR is an effective program in reducing risk factors and increasing both physical capacity and the recurrence of cardiac events in hospitalized patients.^8-10^ CR basically combines stress management interventions with cognitive-behavioral techniques.^2^ However, previous studies suggested that psychological symptoms and subjective stress in post-infarction patients could also be affected by different, but still unknown, variables.^1,11^ Thus, there are some predictive components whose identification could increase the efficacy of treatment interventions. For example, health casual attributions could potentially mediate the stress reduction’s process: indeed, previous reports^12-15^ suggested that both causal attributions and perceived heart risk factors (PHRFs) affect cardiac patients’ health behaviors. Based on these considerations, the present study aimed to assess (i) the effect of CR on patients PHRFs and psychological stress, (ii) how PHRFs difference scores at pre-post CR can affect the changes in patients’ psychological stress.

## Materials and Methods

### Design and context

In this longitudinal study, coronary artery bypass graft (CABG) patients who referred to CR center of Imam Ali hospital of Kermanshah (Iran) from June to November 2016 were assessed. Inclusion criteria included age between 30 and 75 years and no physical limitations to exercise. The exclusion criteria included: presence of severe chest pain or stable dyspnea during aerobic exercises and more than 10% absences during the CR program. In the study period, a total of 138 individuals were admitted to the CR program. Therefore, all of these people entered the study without any sampling. However, 16 patients were excluded due to lack of inclusion criteria and 12 people were excluded due to drop out of the CR session. Therefore, 110 people entered the analysis. Since the study uses regression analysis, the minimum size of the sample was calculated using (N= predictive variables {5} × {8} + 50) formula.^16^ Thus, the minimum sample size should be 90 people. Patients were enrolled in the study after providing written consent. This study was conducted in accordance with the declaration of Helsinki and with ethical guidelines of Kermanshah University of Medical Sciences.

Patients’ demographic (gender, educational level, occupation and marital status) and clinical data (smoking, alcohol and substance abuse, previous history of myocardial infarction, hypertension, diabetes, and hyperlipidemia) were collected by a cardiologist and a clinical psychologist one week before starting CR. Patients’ attendance during the CR program was recorded by nurses. The PHRFs scale^17^ and the stress subscale of Depression, Anxiety, and Stress Scale (DASS-21)^18^ were administered one week before and one week after CR. After an explanation of the study, patients completed the questionnaires individually: in case of illiteracy, a psychologist read the questions and recorded patients’ responses.

### Outpatient CR protocol

CR is an evidence-based 8-week rehabilitation program for cardiovascular outpatients, adapted from the American Heart Association and the American Association of Cardiovascular and Pulmonary Rehabilitation.^19^ CR is administered 3 times a week, for a total of 26 sessions: patients attend 1-hour training and 1-hour exercise sessions: the latter includes a warm-out (10 min), a dynamic exercise (45 min) and a recovery (5 min) phase. The training sessions focus on management of stress, risk factors, healthy nutrition, and weight control. Dynamic exercises included straining movements and running on a treadmill.^10,20^

### Instruments

#### The Perceived Heart Risk Factors Scale (PHRFS)

The PHRFS is a 25-item self-report scale recently developed by Saeidi and Komasi.^17^ It consists of 5 subscales, that evaluates biological (3 items), environmental (5 items), behavioral (6 items), psychological (7 items) and physiological risk factors (4 items). Subscale total scores can be combined into a total score. Each item is rated on a 5-point Likert-type scale (0: never – 4: very great), with higher scores indicating higher perceived risk factors. PHRF showed a moderate to good internal consistency (Cronbach’s alpha for total scale and subscales were 0.93, 0.63, 0.83, 0.82, 0.83, and 0.97, respectively), as well as a good content and construct validity.^17^ In the present study, internal consistency was 0.930.

#### The Depression, Anxiety, and Stress Scale (DASS-21)

This DASS is a 21-item self-report measure of psychological distress, originally developed by Lovibond and Lovibond.^18^ It consists of 3 subscales, which evaluate depression (7 items), anxiety (7 items) and stress (7 items). Each item is rated on a 4-point Likert-type scale (0=did not apply to me at all, 3=applied to me very much, or most of the time). In a study among the Iranian population, Cronbach’s alpha the questionnaire for depression, anxiety, and stress was 0.77, 0.79, and 0.78, respectively.^21^ In addition, this scale had a good concurrent validity with Beck Depression Inventory, Zung Anxiety scale, and Perceived Stress Scale.^21^ Other study reported a good Cronbach’s alpha for the Stress subscale (0.81) in an Iranian sample.^22^ The DASS has a good convergent validity with Beck Depression Inventory.^22^ In the present study, only the Stress subscale was used; the internal consistency was 0.808.

### Statistical analysis

Preliminary analyses were conducted to investigate the presence of outliers and violations of normality assumptions.^16^ In addition, collinearity and multicollinearity were studied. We tested hypothesis 1 of a significant effect of CR on patients’ PHRFs and psychological stress, using paired sample *t* tests. Effect sizes were calculated for each variable using Cohen’s *d*. Finally, we investigated hypothesis 2 of a significant predictive effect of PHRFs difference scores at pre-post CR on decreasing changes in stress scores of the patients (e.g. the correlation between PHRFs difference scores with stress difference score). The main analysis was done using Pearson *r* correlation indexes, and multiple regression analysis. Effect sizes were investigated using R^2^. All analyses were performed using Statistical Package for Social Science (SPSS) version 20. All statistical tests were 2-sided; a *P* value ≤ 0.05 was considered significant.

## Results

The patients mean age was 58.3 ± 9.1 years. Other demographic and clinic data at baseline are reported in Table 1. Table 2 shows means, standard deviations, paired sample t-test results and effect sizes for all variables pre-post CR. Results evidenced that the CR program is effective only in reducing patients’ psychological stress (*P* < 0.05). The CR is not effective in improving PHRFs (*P* > 0.05).

**Table 1:** Demographics and clinical data of the sample

**Variables**	**Total (N = 110)**
Sex, male (%)	65 (59.1)
Marital status (%)	
Single	2 (1.8)
Married	91 (82.7)
Divorced	17 (15.5)
Education (%)	
Middle school or less	76 (69.1)
High school	19 (17.3)
University	15 (13.6)
Job (%)	
Employee	10 (9.1)
Self-employee	31 (28.2)
Housekeeper	42 (38.2)
Retired	27 (24.5)
Smoking (%)	
Never	86 (78.2)
Cessation	23 (20.9)
Active	1 (0.9)
Substance abuse (%)	
Never	102 (92.7)
Cessation	6 (5.5)
Active	2 (1.8)
Alcohol drinking (%)	
Never	106 (96.4)
Cessation	3 (2.7)
Active	1 (0.9)
Hypertension (%)	53 (48.2)
Diabetes (%)	31 (28.2)
Hyperlipidemia (%)	49 (44.5)
Myocardial Infarction history (%)	23 (20.9)

**Table 2:** Means, standard deviations and paired sample *t* tests between pre- and post-CR scores on the main outcome variables (n=110)

**Variables**	**Pre-CR**	**Post-CR**	**Correlation**	**Difference**	***t***	***P*** ** value**	**Cohen's** ***d***
Biological risk factor	5.36 ± 2.39	5.79 ± 2.50	0.251	0.43 ± 3.00	1.495	0.138	-0.144
Environmental risk factor	13.93 ± 4.64	14.18 ± 4.36	0.546	0.25 ± 4.30	0.621	0.536	-0.057
Physiological risk factor	11.73 ± 3.19	11.74 ± 2.84	0.206	0.01 ± 3.81	0.025	0.980	-0.003
Behavioral risk factor	16.47 ± 5.09	17.20 ± 4.57	0.441	0.73 ± 5.13	1.487	0.140	-0.142
Psychological risk factor	20.68 ± 4.11	21.42 ± 4.51	0.572	0.74 ± 4.00	1.930	0.056	-0.185
Psychological stress	9.61 ± 5.09	7.79 ± 4.38	0.639	- 1.82 ± 4.07	4.684	0.001	0.452

As shown in Table 3, difference scores of all risk factors except psychological ones were significantly correlated with changes in stress score (all *P* < 0.05). As regards the regression analysis, the overall model was significant (F=2.596, *P* = 0.030) and it explained 11.1% of the variance for the dependent variable (see Table 3). However, among the independent variables only biological risk factors of PHRF were significant predictors of changes in stress score (β=0.212, *P* = 0.029). The results suggest that for any unit increase in the variable of PHRF biological risk factors, Delta R^2^ in stress increase 0.21 from pre- to post-CR.

**Table 3:** The correlations and multiple regression model for perceived risk factors and stress

**Perceived risk factors**	**Psychological stress**		**B**	**β**	***t***	***P *** **value**
	***r***	***P***				
Biological risk factor	0.236	0.013	0.288	0.212	2.215	0.029
Environmental risk factor	0.208	0.030	0.097	0.102	0.805	0.423
Physiological risk factor	0.228	0.017	0.126	0.118	0.819	0.415
Behavioral risk factor	0.231	0.015	0.070	0.088	0.544	0.587
Psychological risk factor	0.076	0.429	-0.103	-0.101	-0.936	0.352

Summary of the model (R = 0.333; R2 = 0.111; F = 2.596; *P* = 0.030).

## Discussion

The present study was done to assess the effect of a CR program on PHRFs and psychological stress. The second aim was the study of changes of PHRFs at pre-post CR in predicting changes in psychological stress. According to the results, difference scores of all risk factors except psychological ones were indirectly correlated with changes in stress score. In other words, an increase in the patients’ perceptions was associated with reduced stress. In addition, the results indicate that difference scores of PHRFs at pre- to post-CR significantly predicts changes in stress score.

CR is a comprehensive, standardized program for the treatment of cardiovascular patients, which has previously shown its efficacy.^5,6^ As such, American Heart Association strongly suggested its adoption in primary and secondary care settings in order to reach as many patients as possible.^23^ Indeed, CR can improve the overall adjustment to both chronic diseases and disabilities. The purpose of CR is to help the patients to resume physical, social, occupational, and psychological activities appropriately.^20^ In addition, one of the main aims of CR is improving stress management and anxiety symptoms.^1^ Given that most cardiac patients are identified stress as major causes,^24-26^ its management is very important.

Indeed, past reports showed the efficacy of this programs in improving the control over stress.^2,5,6^ Stress is one of the most important psychological risk factors for the development cardiovascular diseases and can affect physical and psychological wellbeing.^22^ Stress and anxiety may be concluded from patients’ perception about unpleasant consequences of the disease. So, CR can play an important role in outcomes of cardiovascular diseases through an increased patient’s sense of control over stress and other psychological symptoms.^20^ Although the CR protocol contained 8 weeks of exercise training and apparently this short term is not enough to control stress, previous trials and review studies show the effect of CR programs in reducing stress during 8-12 weeks.^5,6^

Our results indicate that difference scores of perceived risk factors at pre- to post-CR significantly predict changes in DASS-21 stress scale scores. In accordance with the results, several studies showed a relation between PHRFs and perceived stress or psychological symptoms such as anxiety and depression.^12-15^ These results could be explained taking into account the so-called Self-Regulation Model,^27^ which describes the perception of disease along five dimensions: identification, cause (patient’s perception about the cause), time schedule, outcomes, and the possibility of control/cure, respectively.^28^ Interestingly, the second dimension of the model corresponds to the perceived heart risk factors (PHRFs), organized into 5 categories (biological, environmental, physiological, behavioral, and psychological) by Saeidi and Komasi.^25,26,29,30^ Therefore, based on the model of self-regulation, patients’ knowledge about the causes of their disease concluded from their extensive experiences.^28^ Health behaviors of the patients affected by their cognition.^28^ So, we hypothesize that stress after a cardiac event derivate from the poor perception about the cause of disease. When patients attend CR, they develop a better perception of their own risk factors, probably through an increased sense of mastery on psychological symptoms^20^ and a progressive reduction in stress.

### Limitations and implications

Our study was the lack of a control group and there isn’t evaluation at follow-up. The participants of this study were exclusively CABG patients; therefore future studies should extend these results focusing also on patients with myocardial infarction (MI), *percutaneous coronary intervention* (PCI) and heart failures (HF). In addition, although our CR program included 26 sessions of training and exercise, other short- or long-term protocols^8-10^ (already used in other centers) should be tested. Considering that the study literature was based on the relationship between perceived risk factors and anxiety and depression^13-15^ and it had a less emphasis on psychological stress,^12^ we just chose the stress component among the subscales of the DASS-21. We recommend using other subscales (anxiety and depression) in future studies. It is recommended to use Beck and Hamilton questionnaires in future studies which are specially designed to evaluate distress level of cardiac patients. Given that patients at the beginning of CR are participating in a series of time-consuming and boring interviews along with anthropometric measurements and blood indicators, we use the short questionnaire (DASS-21) to evaluate them. Other clinical tools in this area can be useful.

## Conclusion

One of the most important objectives of CR is improving the psychological symptoms (i.e. stress and anxiety) and improving stress management skills after a cardiac disease. In this context, PHRFs appears to be significant predictive components in reducing stress in CR. So, it is recommended that practitioners focus on patients’ perception of risk factors to facilitate stress management in CR programs.

## Ethical approval

This study was approved by the Ethics Committee of Kermanshah University of Medical Sciences.

## Competing interests

All authors declare no competing financial interests exist.

## Acknowledgment

We appreciate the Clinical Research Development Center of Imam Reza Hospital and Cardiac Rehabilitation Center of Imam Ali Hospital, Kermanshah University of Medical Sciences.
